# Detection of Mitochondrial COII DNA Sequences in Ant Guts as a Method for Assessing Termite Predation by Ants

**DOI:** 10.1371/journal.pone.0122533

**Published:** 2015-04-08

**Authors:** Tom M. Fayle, Olivia Scholtz, Alex J. Dumbrell, Stephen Russell, Simon T. Segar, Paul Eggleton

**Affiliations:** 1 Institute of Entomology, Biology Centre of Czech Academy of Sciences and Faculty of Science, University of South Bohemia, Branišovská 31, 370 05 České Budějovice, Czech Republic; 2 Forest Ecology and Conservation Group, Imperial College London, Silwood Park Campus, Buckhurst Road, Ascot, Berkshire, SL5 7PY, United Kingdom; 3 Institute for Tropical Biology and Conservation, Universiti Malaysia Sabah, 88400, Kota Kinabalu, Sabah, Malaysia; 4 Global Conservation Program, Wildlife Conservation Society, Bronx, New York 10460, United States of America; 5 School of Biological Sciences, University of Essex, Wivenhoe Park, Colchester CO4 3SQ, United Kingdom; 6 Science Facilities Department, Natural History Museum, Cromwell Road, London SW6 5BD, United Kingdom; 7 Life Sciences Department, Natural History Museum, Cromwell Road, London SW6 5BD, United Kingdom; University of Guelph, CANADA

## Abstract

Termites and ants contribute more to animal biomass in tropical rain forests than any other single group and perform vital ecosystem functions. Although ants prey on termites, at the community level the linkage between these groups is poorly understood. Thus, assessing the distribution and specificity of ant termitophagy is of considerable interest. We describe an approach for quantifying ant-termite food webs by sequencing termite DNA (cytochrome c oxidase subunit II, COII) from ant guts and apply this to a soil-dwelling ant community from tropical rain forest in Gabon. We extracted DNA from 215 ants from 15 species. Of these, 17.2 % of individuals had termite DNA in their guts, with BLAST analysis confirming the identity of 34.1 % of these termites to family level or better. Although ant species varied in detection of termite DNA, ranging from 63 % (5/7; *Camponotus* sp. 1) to 0 % (0/7; *Ponera* sp. 1), there was no evidence (with small sample sizes) for heterogeneity in termite consumption across ant taxa, and no evidence for species-specific ant-termite predation. In all three ant species with identifiable termite DNA in multiple individuals, multiple termite species were represented. Furthermore, the two termite species that were detected on multiple occasions in ant guts were in both cases found in multiple ant species, suggesting that ant-termite food webs are not strongly compartmentalised. However, two ant species were found to consume only *Anoplotermes*-group termites, indicating possible predatory specialisation at a higher taxonomic level. Using a laboratory feeding test, we were able to detect termite COII sequences in ant guts up to 2 h after feeding, indicating that our method only detects recent feeding events. Our data provide tentative support for the hypothesis that unspecialised termite predation by ants is widespread and highlight the use of molecular approaches for future studies of ant-termite food webs.

## Introduction

Termites and ants are ubiquitously abundant in tropical rain forests around the world [1–3], with both groups being functionally important [4]. Termites act to break down organic matter, feeding on and processing a range of materials, from lichens, mosses and algae to dead leaves, dead wood, humus, and even soil [5,6]. Ants fill a wider variety of niches, for example as predators, “cryptic” herbivores, and seed dispersers [7]. Given that many species of ant are at least partly predatory and termites are potential prey, it has always been assumed that ants eat large numbers of termites and therefore, that the ant-termite trophic interaction is a key one in tropical rain forests [1,8]. This assumption is supported by both the numerous studies focussing on particular ant or termite taxa [8], and the remarkable diversity in defence strategies employed by termite soldiers, which may have evolved in response to predation pressure from ants [9,10]. For example, some species of ant, including those from the genera *Centromyrmex* [11–13], *Megaponera* [12,14–16], *Anochetus* [17], *Tetramorium* [18], and *Paltothyreus* [19] specialise on particular termite taxa, while species from a wide range of genera are known to predate termites opportunistically to a greater or lesser extent [12]. Other species, such as *Dorylus* (*Anomma*) driver ants actively avoid termites, only feeding on alates during swarming [20]. Furthermore, there is substantial (correlational) evidence that the nest density of termites is limited by the abundance of both dominant [21–23] and non-dominant ant species [24]. However, although many studies have confirmed the existence of the interaction between individual ant and termite species, or between small subsets of communities [11,12,17], we know of no direct evidence for the importance of this relationship across entire communities of ants and termites. This is mainly because the majority of ant-termite predation events occur underground, making them difficult to observe.

Understanding the strength of the linkages between these two ecosystem engineers is important, as it is likely to affect many other processes. For example, predation of termites by ants is expected to limit termite-mediated decomposition of organic matter [23,25], even where ants are not termite specialists [23]. Furthermore, since ants are important predators of groups other than termites, increases in the abundance of termites might limit these other taxa through apparent competition e.g. [26].

A method is therefore required by which ant predation on termites can be inferred for whole communities. Numerous laboratory feeding trials have been conducted [27–29], but this approach requires live collection of a complete set of termite species likely to be encountered in the wild, a requirement that also limits field-based feeding trials. Active labelling, for example with tracers such as rabbit immunoglobin protein, can be used when large numbers of replicates are not required, and where candidate colonies of termites can be fed with labelled food items [30]. However, for community-level analyses this method would be highly labour intensive, particularly for subterranean termites, since it would require feeding every termite colony in an area with labelled food.

We propose molecular methods as the solution to these challenges in generating community-level data on ant-termite interactions. Such methods have been used to great effect to reveal previously cryptic species interactions, including parasitism [31,32], herbivory [33] and predation [34]. Specifically, we investigate the potential for sequencing entire individual ants, including their gut contents, as a method for determining the rates and specificities of ant predation on subterranean termites. Note that adult ants usually feed only on liquid food, while solids are fed to larvae [35]. However, previous work using protein marking of termites has demonstrated that termite remains are detectable in adult ants (both workers and queens) [30]. Hence our expectation is either that adult ants feed on termite haemolymph, or that sufficient termite DNA remains from the ant having carried a termite (but not having ingested it).

## Materials and Methods

Field collections were made from primary rain forest in Ivindo National Park, Gabon (S 00°10’ E 12°32’). Field work was carried out between March and July 2008 by OS. Permits and approvals were obtained for field work from ANPN (Agence National Des Parcs Nationaux), and CENAREST (Centre National de la Recherche Scientifique et Technologique). A total of 2,304 soil cores were excavated (25x25 cm area, 10 cm depth) over a 1 ha area, and representatives of all ant and termite species present in each core were collected into 95% ethanol, and preserved at -70°C on return from the field (ranging from 1–5 months later). Preliminary analyses demonstrated a spatial association between the termite and predatory ant communities, indicating a possible trophic interaction in the assemblage [36]. We selected 255 ants at random from the occurrences across the entire grid, with no two ants coming from the same soil pit (note that individuals from rare species were not used for all statistical analyses). Ants were randomly selected because we wanted to test for overall community patterns in termite predation, rather than focussing on a particular subset of ant species. These ants were then identified initially based on external morphology.

We then extracted DNA from entire individual ants. Specimens were dry-ground with a few grains of sand using a micro pestle in 1.5 ml Eppendorf tubes, with 180 μl ATL lysis buffer (Qiagen Ltd, Crawley, UK) and 20 μl Proteinase K [40 U/ml] (Sigma) and left to digest overnight at 55°C with agitation. Standard Qiagen blood and tissue kits were used to perform extractions on the lysates, following manufacturer’s instructions (Qiagen Ltd). The concentration of DNA in each extract was recorded using a Nanodrop 8000 DNA spectrophotometer (Thermo Fisher, Waltham, MA USA) to measure the amount of template being added to the PCR reaction. We then conducted PCR on the resulting DNA using termite-specific cytochrome c oxidase subunit II (COII) primers (Sequences, 5’ to 3’: *Modified A-tLeu*: CAGATAAGTGCATTGGATTT, *B-tLys*: GTTTAAGAGACCAGTACTTG; [37]). We used COII, rather than the more commonly-used COI, because the number of available termite sequences is greater for the former. To assist in delineating ant species, extracted DNA was also used for another PCR using universal primers for cytochrome c oxidase subunit I (COI) *LCO1490*: 5’-GGTCAACAAATCATAAAGATATTGG-3’ and *HCO2198*: 5’-TAAACTTCAGGGTGACCAAAAAATCA-3’ [38]. Individual PCR reactions were carried out in a 25 μL reaction volume comprising: 16.5 μl PCR grade Water, 2.5 μl 10x reaction buffer (supplied with TAQ), 1.5 μl Magnesium chloride [50 mM], 1 μl dNTP mix [100mM], 1 μl of each primer [10 μM], 0.5 μL Hi fidelity BIOTAQ (Bioline, London, UK), and 1 μL of DNA template [2–8 ng/μl]. PCR conditions for reactions utilising primers *Modified A-tLeu/B-tLys* were: 94.0°C for 5 min; 35 cycles at 94.0°C for 30 sec, 53°C for 30 sec and 72.0°C for 1 min; and 72.0°C for 10 min. PCR conditions for reactions utilising primers *LCO1490/HCO2198* were: 94.0°C for 5 min; 40 cycles at 94.0°C for 30 sec, 48°C for 30 sec and 72.0°C for 1 min; and 72.0°C for 10 min. All reactions were conducted using a Techne TC-512 thermo cycler (Bibby Scientific, Stone, Staffordshire, UK). The resulting ca. 900 base pair PCR products were purified using a purification plate method (Merck, Millipore). DNA strands in the forward and reverse direction were sequenced on an ABI 3730XL capillary DNA analyser (Applied Biosystems Paisley, UK), using Big Dye Terminator kit version 1.3 (Applied Biosystems.) following manufacturer’s instructions, conducted at the Natural History Museum London sequencing facility. Text resulting from the AB1 chromatogram traces was edited by hand using FinchTV v1.4.0 (software from Geospiza Inc, 2006) and poor quality sequences were excluded. Contigs were assembled using Unipro UGENE v.1.13.1 [39]. All sequences have been archived in the European Molecular Biology Laboratory (EMBL) data base (European Nucleotide Archive, accession numbers LN608994-LN609187; http://www.ebi.ac.uk/ena/data/view/LN608994-LN609187).

In order to measure the specificity of ant-termite interactions we clustered COII sequences obtained from ant guts into molecular operational taxonomic units (MOTUs) using jMOTU v.1.0.7 [40]. This method provides an objective way of defining the cut-off point that should be used to delimit intra- and inter-specific variation, and can be used in combination with phylogenetic assignment of MOTUs to provide more robust delimitation of MOTUs. Given that many of the species involved were sequenced here for the first time, the lack of a termite species list for the area, and the poor state of termite species-level taxonomy in the region, a full reference library for grouping sequences with known species was unavailable. We set the low BLAST identity filter to 97 but otherwise the settings were left as default. We clustered ant sequences in the same way. For both taxa we visually identified the percentage cut-off points that corresponded to the ‘barcode gap’ [41] (although note that animal barcoding commonly refers to the COI locus, not the COII locus). This can be seen in a plot of MOTU number vs. percentage cut-off and is the value over the short plateau in MOTU number directly following the sharp decrease resulting from intra-specific clustering. For ants this was a pair-wise difference of 2.5% and for termites 2.0%. Sequences were fully aligned in ClustalX v2.1 [42] using the default settings. Bootstrapped maximum likelihood phylogenies were generated using the web-server edition of RAxML [43]. For both ants and termites we selected a General Time Reversible Model including a Gamma distribution of rate variation between sites and a proportion of invariant sites (GTR+I+G). Where available we included published sequences from ant and termite con-specifics and appropriate out groups [37,44–47]. To test for differences in frequency of termite consumption between different ant taxa (not accounting for termite identity), we used Fisher’s exact tests on frequencies of detection vs. non-detection (P-values generated using 10^6^ simulations). Tests were conducted at the ant species level, genus level, and subfamily level. To visualise ant-termite interactions, for the ants for which their gut content could be assigned to termite MOTUs, we plotted a bipartite food web using the *bipartite* package in R [48].

In order to assess limits of detection time following ingestion of termites by ants we conducted a laboratory feeding experiment. A single colony of *Camponotus* sp. was fed *ad libitum* live *Reticulitermes santonensis*, and collections of ants made at a range of times following feeding (0 h, 1 h, 2 h, 4 h, 8 h, 16 h, 32 h and 64 h, N = 2 for each time). We also collected ants that had recently been seen to carry termites, control (unfed) ants prior to feeding, and living termites. We then dissected out the contents of each ant gaster (including the crop), and extracted total DNA using standard Qiagen blood and tissue kits following manufacturer’s instructions (Qiagen Ltd). We then conducted PCR using the termite specific primers in the same manner as detailed above, and ran agarose gels to screen for presence of termite COII sequences.

## Results and Discussion

Using combined morphological and molecular determinations of field specimens, we identified 39 ant species from 18 genera (S1 Fig). We present here only data for the 15 ant species from 13 genera for which the number of individuals was >5, with the exception of three species with fewer individuals that were included due to a known specialisation on termites (215 individuals in total, Table 1). For morphological species for which cryptic clades were detected, but for which not all individuals were sequenced, we retained morphological species definitions, because it was not possible to assign unsequenced individuals to molecularly defined clades. We observed positive PCR amplifications using termite COII primers from 37 of the 215 ant specimens, a 17.2% detection rate (Table 1). This indicates that sufficient termite DNA remains associated with adult ants for amplification, despite workers usually only carrying termites, or perhaps drinking their haemolymph. This persistence of termite prey material associated with worker ants has previously been demonstrated using protein marking [30]. The species with the highest proportion of detections was *Camponotus* sp. 1, in which 5/7 workers tested positive for termite DNA, while the species with the lowest detection rate was *Ponera* sp.1, with 0/7 detections. However, despite these differences between ant species in the proportion of termite “hits”, detections were not found to be significantly heterogeneously distributed between species (Fisher’s exact test: N = 15, P = 0.557), genera (N = 13, P = 0.476) and subfamilies (N = 4, P = 0.851). On the basis of the current evidence (with a relatively small number of samples), we are unable to conclude that there are any differences between ant taxa in terms of their tendency to prey on termites (although note that there might still be preferences between ant species for particular termite taxa, see below). This agrees broadly with previous observations that a wide range of ant genera are known to predate termites both opportunistically and as specialists on particular termite species [12]. However, the fact that the single species from the genus *Centromyrmex*, which is known to be a termite specialist predator [11–13], only had a 10% detection rate cautions against using detection rates as a direct measure of termite predation strength. This point is further demonstrated by the low hit rates for the two *Odontomachus* species, both of which are thought to predate large numbers of termites [49,50].

**Table 1:** Species-level detection rates for termite DNA in ant guts.

Genus	Morphospecies no.	Species name	Individuals tested	Termite detections
*Anoplolepis*	1	*carinata*	35	4
*Camponotus*	1		7	5
*Centromyrmex*	2		10	1
*Crematogaster*	1		22	2
*Dorylus*	10*		9	1
*Euponera*	3*	*brunoi*	30	5
*Hypoponera*	1		2	1
*Leptogenys*	2		6	2
*Mesoponera*	1	*caffraria*	44	8
*Mesoponera*	5*	*senegalensis*	6	1
*Odontomachus*	1	*troglodytes*	3	1
*Odontomachus*	2	*assiniensis*	4	1
*Pheidole*	3*	*pulchella*	13	2
*Ponera*	1		7	0
*Tetramorium*	8*		17	3
**Total**			**215**	**37**

Only ant species with >5 individuals presented here, with the exception of known termite predators in the genera *Hypoponera*, and *Odontomachus*.

*Ant species for which multiple cryptic molecular clades were present, but for which no morphological correlates were found. Since not all ant individuals were sequenced, the morphological identifications for these species were retained.

**Fig 1:**
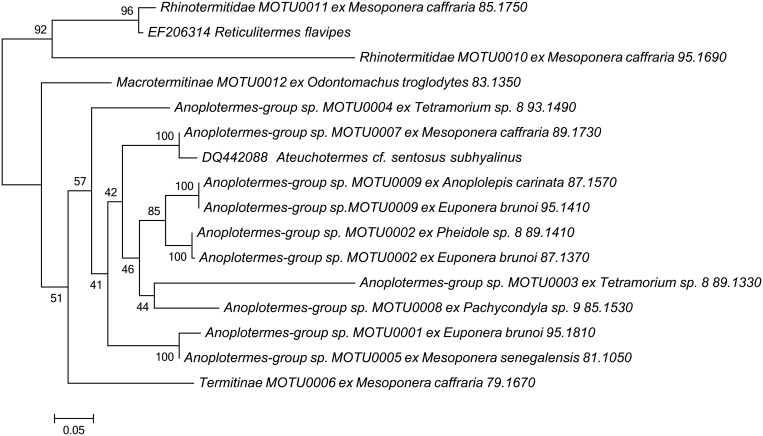
Maximum likelihood phylogeny of termites consumed by ants based on COII sequences. Phylogeny rooted to Rhinotermitidae. Node values give bootstrap support. Scale bar represents substitutions per site based on the GTR+I+G model.

**Fig 2:**
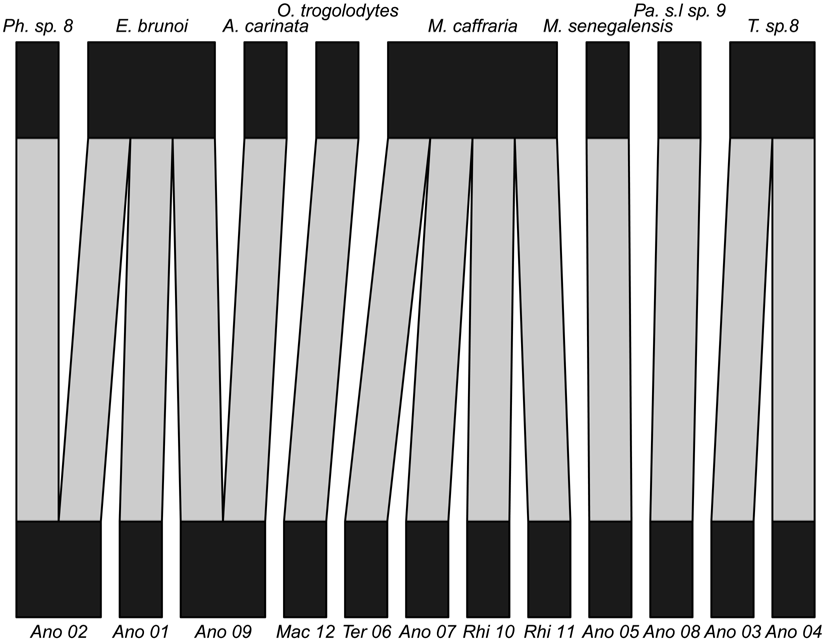
Bipartite hypogeic (below soil-surface) food web visualising ant predation on termites in rain forest in Gabon. For termites (lower level), abbreviations are as follows. Ano = *Anoplotermes* group; Mac = Macrotermitinae; Ter = Termitinae; Rhi = Rhinotermitidae. For ants, see Table 1 for full genus names. Even with this small dataset there are three species of ants that prey on more than one termite species, and two termite species that are preyed on by multiple ant species. Note that *Pheidole* sp 8 is included here, although it was not included in statistical analyses, since we tested fewer than six ant individuals for this species.

From the 37 termite PCR products sequenced, we found 14 were of suitable quality for further analysis. These clustered into 12 MOTUs that gave termite matches of over 80% when compared to sequences deposited in Genbank [51] using the discontiguous BLAST algorithm [52] which is suitable for cross-species comparisons when the differences between query and references sequences is less than 95% (as is the case for 12/14 of the termite sequences). A large proportion of the termite sequences were from the soldierless termite clade (the *Anoplotermes*-group; Termitidae: Apicotermitinae; Fig 1) that are abundant members of the local termite community in the soil [3]. The other close BLAST matches were with groups that are commonly found in the soil except for the Rhinotermitidae, which are rarely encountered in the soil in African old growth tropical rain forests [53]. These are wood-feeding termites and were probably devoured earlier in dead wood. Given the small total number of positive termite identifications and the high diversity of the system, even if there is no species-specificity at all, one would expect multiple “singleton interactions”, which is what we observed (three instances; Fig 2). The only informative interactions in terms of detecting species-level specificity of ant-termite predation are those in which there are multiple records of one ant species consuming termites. Multiple individuals with identifiable termite DNA were found in three species of ants (*Euponera brunoi*, N = 3; *Mesoponera caffraria*, N = 4; *Tetramorium* sp. 8, N = 2), with all three predating multiple termite species. This provides no evidence for termite species-level specificity in ant predation. However, both *E*. *brunoi* and *Tetramorium* sp. 8 were only found to feed on termites from the *Anoplotermes* group, indicating a possible genus-group level specialisation on this soil feeding group.

From the ants that were experimentally fed on termites, we detected PCR products of the correct length to be termite COII after 1 h (1/2 samples), and 2 h (1/2 samples; S2 Fig). There was also a very weak band after 64 h (1/2 samples), although we interpret this with caution, due to the lack of any detections at intermediate times. An ant that had been recently observed to carry a termite showed a strong COII-length band, as did a whole termite (1/1 sample for each). These results indicate that fragments of COII sufficient in length to be detected using PCR remain in ant guts up to 2 h after feeding. Although feeding events occurring >2 h previously are not likely to be detected, we argue that the relatively high percentage of occurrences of termite sequences in wild ant guts (17.2%) indicates that where it occurs, ant feeding on termites seems to be sustained through time, hence validating the use of this method for constructing ant-termite predation food webs. If our primers amplify COII with differing efficiency from different termite species, or if amplification is more difficult for particular ant species, then this might cause biases in our estimations of the strengths of different interactions. Hence we are able to confidently state the presence of particular predatory interactions between species, but the absence of an interaction in our data (and in other data generated using this method) should be interpreted with caution. Furthermore, note that our method is unable to differentiate between ant predation on termites, and ant scavenging on termites that are already dead, although presumably the latter is less likely, since degradation of termite DNA will be more advanced for individuals that are dead prior to consumption.

With the proviso that the number of identified sequences was small, our results indicate that termite predation by ants may be widespread, with ant species not differing in the frequency of termite consumption, and with tentative support for both generalism, and specialism at the level of the termite genus-group for ant predators. Our work shows that COII sequencing is a viable method for detecting ant predation of termites. The use of this method, applied to specimens from field collections and laboratory feeding assays, should be expanded to larger datasets, in order to shed more light on the nature of interactions between these key ecological groups.

## Supporting Information

S1 Fig Maximum likelihood phylogeny of the ants sampled that provided high quality COI sequences. Phylogeny rooted to *Leptanilla* sp. and *Protanilla* sp. Node values give bootstrap support. Scale bar represents substitutions per site based on the GTR+I+G model. Note that three ant species were not sequenced, only morphologically determined, and so these are not included in the phylogeny.
(TIF)

S2 Fig Agarose gel of PCR products from experimental feedings of *Camponotus* sp. ants on live *Reticulitermes santonensis*. Numbers in hours refer to time after feeding (N = 2 for each time). TM = the head of an ant that was observed to carry a dead termite prior to collection. A = unfed ant. T = termite. C = PCR negative control. White arrows indicate bands at the expected length for COII. Note that the band at 64 hours is very faint.
(TIF)
