# Supplementary figures and images for: Detection of Mitochondrial COII DNA Sequences in Ant Guts as a Method for Assessing Termite Predation by Ants
Source: PLoS One. 2015 Apr 8;10(4):e0122533. doi: 10.1371/journal.pone.0122533 (PMC4390358; doi:10.1371/journal.pone.0122533)

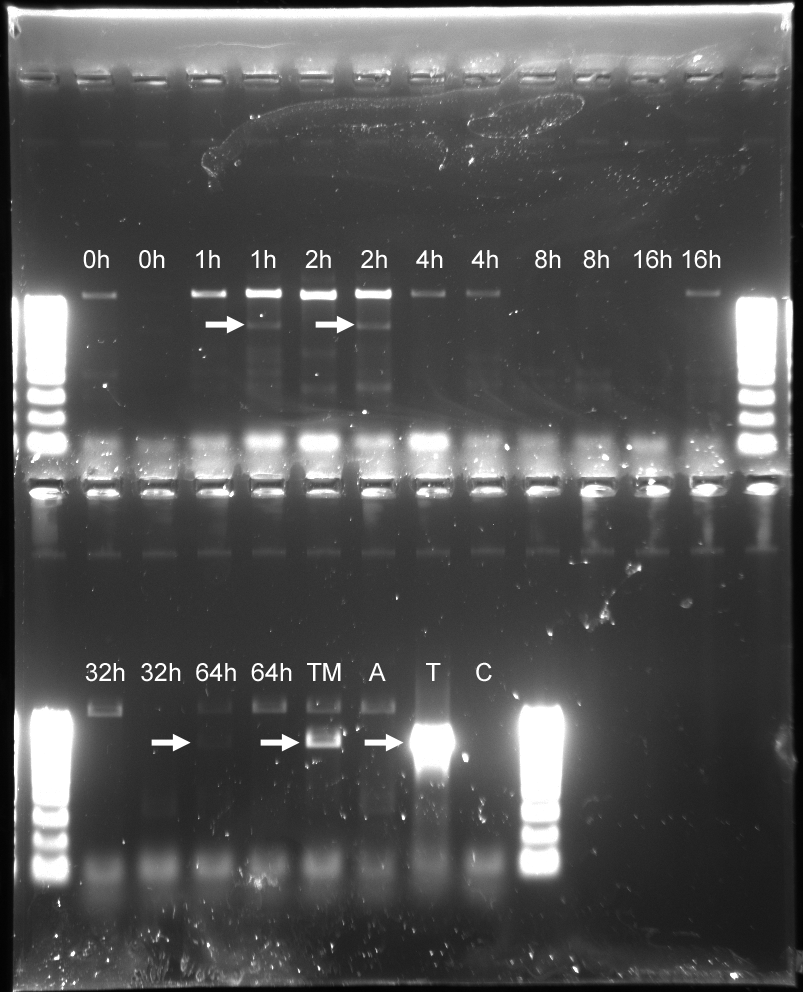

Supplement: S2 Fig — Numbers in hours refer to time after feeding (N = 2 for each time). TM = the head of an ant that was observed to carry a dead termite prior to collection. A = unfed ant. T = termite. C = PCR negative control. White arrows indicate bands at the expected length for COII. Note that the band at 64 hours is very faint. (TIF) [file pone.0122533.s002.tif]
